# The Impact of Big Five Personality Traits on HPV Vaccination Willingness Among Female Healthcare Undergraduates: A Cross-Sectional Study in Chengdu, China

**DOI:** 10.3390/vaccines14070610

**Published:** 2026-07-11

**Authors:** Min Xie, Shan Lai, Jing Lei, Qin Feng, Qiong Liang, Miao Zhao

**Affiliations:** 1 Department of Cosmetic Plastic and Burn Surgery, The First Affiliated Hospital of Chengdu Medical College, Chengdu 610500, China; 2 College of Pharmacy, Chengdu Medical College, Chengdu 610500, China

**Keywords:** Big Five personality traits, HPV vaccine, vaccination willingness, female undergraduates, healthcare majors

## Abstract

*Background and Objectives:* HPV vaccination is critical for cervical cancer prevention, but HPV vaccination coverage stays low among young women in China. While various factors shaping vaccination willingness have been explored, the role of personality traits remains insufficiently understood. This study investigated the relationships between Big Five personality traits and three facets of HPV vaccination willingness (consideration, determination, recommendation) among female healthcare undergraduates. We also specifically examined whether academic major moderated the association between extraversion and vaccination willingness. *Methods:* A cross-sectional survey was conducted among 703 female undergraduates enrolled in healthcare majors at a medical college in Chengdu using a stratified cluster sampling method. Data were collected via online questionnaires, including the 10-item Big Five Inventory (BFI-10) and a scale measuring three motivational facets of HPV vaccination willingness: consideration, determination and recommendation. Descriptive statistics, correlation analysis and hierarchical regression analysis were performed to examine the research questions. *Results:* A total of 672 valid questionnaires were analyzed. Distinct associations were found between personality dimensions and each facet of vaccination willingness. Agreeableness (*β* = 0.193, *p* < 0.01) and openness (*β* = 0.079, *p* < 0.05) correlated with vaccination consideration (ΔR^2^ = 0.061). Extraversion (*β* = 0.103, *p* < 0.05), agreeableness (*β* = 0.113, *p* < 0.01) and conscientiousness (*β* = 0.096, *p* < 0.05) correlated with determination (ΔR^2^ = 0.049). Extraversion (*β* = 0.125, *p* < 0.01), agreeableness (*β* = 0.129, *p* < 0.01), conscientiousness (*β* = 0.102, *p* < 0.01) and neuroticism (*β* = 0.081, *p* < 0.05) were positively related to recommendation willingness (ΔR^2^ = 0.061). Academic major did not exert a significant moderating effect. *Conclusions:* The findings highlight the complexity of the relationships between personality traits and vaccination attitudes and behaviors. While the observed associations were modest in magnitude, this study provides preliminary empirical evidence that may inform personality-sensitive HPV vaccination promotion strategies. Tailored, multi-dimensional interventions that consider diverse population characteristics could be considered to optimize HPV vaccination outreach, though their effectiveness awaits further validation.

## 1. Introduction

Cervical cancer is the fourth-most common malignancy threatening women’s health globally [1]. Human papillomavirus (HPV) is responsible for nearly all cases of cervical cancer [2]. Accumulated evidence has confirmed that HPV vaccination effectively prevents HPV infection and substantially reduces cervical cancer incidence [3,4]. However, current HPV vaccination coverage in China remains remarkably low. A 2025 survey of over 4000 Chinese female college students reported an 18.11% vaccination rate and a 40.19% willingness to vaccinate [5]. Therefore, identifying factors influencing HPV vaccination willingness is essential to support targeted vaccination promotion.

While prior studies identify sociodemographic factors (e.g., age, academic major, HPV knowledge) as key predictors of HPV vaccination willingness [6,7,8,9,10], stable psychological traits—such as personality—remain understudied relative to contextual variables. Personality traits have been extensively demonstrated to impact health-related decisions and behaviors [11,12,13,14]. The Big Five personality model, consisting of extraversion, agreeableness, conscientiousness, neuroticism and openness, is the most widely adopted framework for studying personality effects on health behaviors, including vaccination [15,16,17]. A recent systematic review by Bleidorn et al. (2025) indicated that most studies found extraversion and agreeableness to be positively associated with positive vaccination attitudes and behaviors [18]. In contrast, findings regarding conscientiousness, openness, and neuroticism were largely inconsistent [18]. Despite these insights, critical gaps persist. Most studies focus on COVID-19, influenza and other common vaccines rather than HPV, rely on unidimensional measures of willingness, and predominantly sample Western populations. Importantly, the HPV vaccine differs from vaccines for acutely threatening infectious diseases in several psychologically relevant aspects: it is primarily targeted at young women, for whom health decisions are often shaped by peers and partners; it is uniquely subjected to social stigmatization and cultural taboos surrounding female sexuality, particularly in non-Western contexts; and it prevents a future cancer risk rather than an immediate health threat, engaging different psychological mechanisms of risk perception and valuation of future health benefits. These fundamental differences underscore the need for dedicated investigations into the personality correlates of HPV vaccination willingness, rather than relying solely on inferences drawn from the broader vaccine literature. Collectively, there is a need for further research to clarify the multi-faceted relationships between Big Five traits and HPV vaccination willingness among targeted populations.

Healthcare undergraduates represent a critical study population, as their vaccination attitudes can significantly influence public health perceptions. Although Yang et al. (2024) reported high HPV vaccination willingness (85.5%) among Chinese female medical students [19], there remains room for improvement. Investigating this group allows us to determine whether personality traits independently influence vaccination decisions within a context of relatively sufficient medical knowledge.

Notably, personality traits are not uniformly distributed across academic disciplines. A systematic review by Vedel (2016) documented consistent personality differences across academic majors, with medical students scoring relatively high on both extraversion and agreeableness compared to students in several other fields [20]. In the present study, a preliminary analysis confirmed that extraversion was the only Big Five trait showing significant between-major differences. It is therefore plausible that the relationship between extraversion and vaccination willingness may not be uniform across academic majors. If extraversion facilitates vaccination willingness partly through social engagement and peer communication, this effect might be amplified or attenuated depending on the social and professional norms of a student’s specific discipline.

This study addresses critical gaps regarding the limited exploration of personality in HPV vaccination, the reliance on unidimensional willingness measures, and cultural barriers in China. By examining the main effects of Big Five personality traits on HPV vaccination willingness and exploring whether academic major moderates the extraversion–willingness relationship, we aim to elucidate independent psychological determinants of HPV vaccination willingness among medical undergraduates. These findings may inform targeted interventions to promote vaccine uptake in this population.

**Figure 1:**
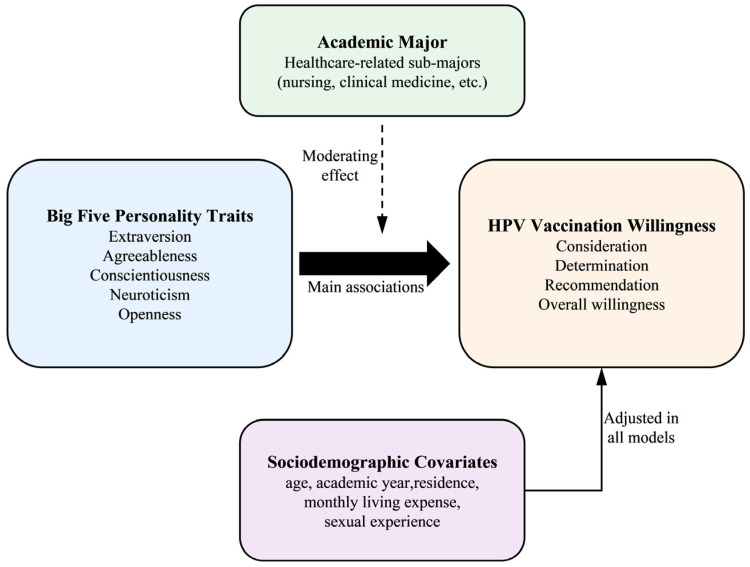
Conceptual framework illustrating the hypothesized relationships among Big Five personality traits, academic major, and multi-dimensional HPV vaccination willingness among female healthcare undergraduates. Solid arrows indicate hypothesized main associations, and the dashed arrow indicates the hypothesized moderating effect of academic major. All regression models were adjusted for sociodemographic covariates.

The conceptual framework of this study is illustrated in Figure 1.

## 2. Materials and Methods

### 2.1. Study Design

This cross-sectional investigation took place from March to April 2024 at a public independent medical college located in Chengdu, Sichuan, China. An electronic questionnaire, accessible via a quick response (QR) code, was used to collect data through the Wenjuanxing platform (https://www.wjx.cn/, accessed on 20 January 2024). The eligible participants were female undergraduate students enrolled in health science programs at the college throughout the study period. The study was conducted in accordance with the Strengthening the Reporting of Observational Studies in Epidemiology (STROBE) guidelines.

### 2.2. Sampling and Participants

Stratified cluster sampling was conducted among undergraduate students of this college. The stratification was based on academic major (Nursing, Clinical Medicine, Pharmacy, and Preventive Medicine) and academic year (first-year, second-year, and third-year), yielding 12 strata in total. Within each stratum, two intact classes were randomly selected, with the exception of the Nursing program, where one class per academic year was selected to avoid overrepresentation of this major in the final sample, given its considerably larger female student enrollment relative to the other three majors. All female students in the selected classes were invited to participate.

The sample size estimation was conducted with reference to prior literature, and the calculation formula is presented as follows:

$$n=\frac{Z_{\alpha /2}^{2}\times p\left(1-p\right)}{d^{2}}\;(\alpha =0.05,Z_{\alpha /2}=1.96)$$

In the above formula, d denotes the permissible error (set to 0.03), and *p* represents the anticipated proportion of female college students willing to accept the HPV vaccine. As documented in a previous Chinese study [8], 96.2% of medical college students expressed willingness to undergo HPV vaccination. We conservatively assigned *p* = 0.9 for sample size computation, with the initial minimum sample calculated as 385. After accounting for a 15% allowance for invalid questionnaires, the final minimum sample size was determined to be 443.

Eligible participants needed to fulfill two inclusion criteria: being Chinese female first- to third-year undergraduates, and having heard of HPV and HPV vaccines. Exclusion criteria included a history of HPV vaccination, as well as severe medical conditions contraindicating HPV immunization. We excluded students with a history of HPV vaccination because our study aimed to examine factors associated with vaccination intention among unvaccinated females, and previously vaccinated individuals could not provide valid data reflecting unresolved vaccination decisions.

### 2.3. Instruments

#### 2.3.1. Demographic Characteristics Questionnaire

Data on demographic characteristics were collected via a self-designed questionnaire, covering key indicators: age, undergraduate major, academic year (1st–3rd year), residential location (urban/rural), monthly living expenses (CNY), and sexual experience (yes/no).

#### 2.3.2. Brief Version of the Big Five Personality Inventory

Personality was assessed using the 10-item Big Five Inventory (BFI-10). This brief measure was originally developed and validated by Rammstedt and John (2007) [21] to efficiently capture personality traits with minimal item burden. In its standard format, the BFI-10 contains 10 items, with 2 items assigned to each of the five dimensions: extraversion, agreeableness, conscientiousness, neuroticism, and openness. Given that agreeableness represents a core independent variable in this study, and considering the developers’ caution that the two-item agreeableness subscale exhibits relatively weak psychometric performance compared to the other four domains, we adopted their recommendation to incorporate a third, supplementary agreeableness item. Specifically, Rammstedt and John (2007) noted that the agreeableness subscale showed the most substantial measurement loss among the five dimensions and advised that researchers for whom agreeableness is a core construct should add an additional item to improve reliability [21]. Accordingly, the instrument used in this study contained 11 items. All items were scored on a 5-point Likert scale (1 = “strongly disagree” to 5 = “strongly agree”). Items 1, 3, 4, 5, and 7 were reverse-scored. After recoding, the scores for each dimension were calculated as the sum of their respective items. Scores for extraversion, conscientiousness, neuroticism, and openness ranged from 2 to 10, while the agreeableness score could range from 3 to 15, with elevated scores reflecting a greater expression of the respective trait.

In the present study, the Cronbach’s α coefficients for each BFI-10 subscale were: 0.58 (extraversion), 0.41 (agreeableness), 0.53 (conscientiousness), 0.55 (neuroticism), and 0.48 (openness). Although these values fell below the conventional 0.70 internal consistency threshold, such estimates are widely accepted for ultra-brief personality measures, and align with prior BFI-10 findings across global [22,23,24] and Chinese [25,26] samples. Two features of the scale account for this: first, α is mathematically constrained by the small number of items (two per trait); second, the items were intentionally chosen to capture broad, heterogeneous aspects of each dimension rather than narrowly overlapping content. Consequently, some authors have argued that α is an unsuitable index for two-item scales [27,28], and several studies have reported adequate validity without relying on α [21,29,30,31]. To further evaluate the reliability of the BFI-10 in this study, supplementary indicators were calculated, including inter-item correlations and Spearman–Brown coefficients. The inter-item correlations were 0.41 (extraversion), 0.18 (agreeableness), 0.14 (conscientiousness), 0.38 (neuroticism), and 0.13 (openness). According to Briggs and Cheek (1986), the optimal range for inter-item correlations in two-item scales is 0.20–0.40 [32]. The values for extraversion and neuroticism fell within this range, whereas those for the other three dimensions were lower, likely reflecting the deliberate selection of heterogeneous items to maximize the breadth of content coverage with minimal items. The Spearman–Brown coefficients were 0.61 (extraversion), 0.52 (agreeableness), 0.57 (conscientiousness), 0.57 (neuroticism), and 0.51 (openness). The developers of the BFI-10 regard it as a psychometrically adequate instrument when time constraints preclude longer measures [21]. Given the practical limitations of class-break administration in this exploratory study, the BFI-10 remains acceptable for preliminary analysis, though the low internal consistency is a limitation, and future work should employ more comprehensive personality assessments.

#### 2.3.3. HPV Vaccination Willingness Scale

Vaccination willingness was measured with the HPV Vaccination Willingness Scale. This scale was originally developed and validated by Gong (2022) [33]. The scale captures three motivational facets—consideration, determination, and recommendation—each represented by a single item: “I will consider receiving the HPV vaccine”, “I will definitely receive the HPV vaccine”, and “I will recommend the HPV vaccine to my friends or classmates”. All items were scored on a 5-point Likert scale (1 = “strongly disagree” to 5 = “strongly agree”). The overall willingness was calculated as the composite score (range 3–15) of the three facets, with higher scores indicating stronger vaccination willingness. In the original validation study, the Cronbach’s α coefficient for this scale was 0.97 among female non-medical college students [33]. In the present study, the Cronbach’s α coefficient for this scale was 0.87, indicating good internal consistency.

Although each facet was assessed using only a single item, this design was intentional to minimize questionnaire length and respondent fatigue. This allows for a better capture of the three conceptually distinct aspects of vaccination willingness, particularly when time is limited. Single-item measures are considered acceptable when the construct of interest is concrete, unidimensional, and clearly defined [34]. The three facets—consideration, determination, and recommendation—each represent a specific, unambiguous motivational state, and have been shown to form a reliable composite scale in prior validation work [33]. Moreover, the present study examined both the individual facets and the overall willingness score, allowing for nuanced insights while mitigating the limitations of single-item measurement.

The full questionnaire is provided as an example in the Supplementary Materials.

### 2.4. Data Collection

The survey was conducted during class breaks without any compensation for participation. First, male students were asked to leave the classroom. The investigators then outlined the study purpose and the inclusion/exclusion criteria to the remaining female students; those who met the inclusion criteria were asked to stay, while those who met any exclusion criterion were requested to leave. Subsequently, the participants were given instructions on completing the questionnaire and were encouraged to ask questions, with the investigators providing additional explanations as needed. After confirming that no further questions remained, the questionnaire was immediately distributed on-site using a QR code. Participants voluntarily scanned the QR code with their personal mobile phones and completed the questionnaire anonymously on the spot. To prevent duplicate entries, only one submission per mobile IP address was allowed. Finally, participants submitted the questionnaire and left the classroom in an orderly fashion.

Invalid questionnaires were defined as those with contradictory personal information or identical answers across all items. Of the 703 distributed questionnaires, all were retrieved. Following the removal of 31 invalid cases, 672 valid questionnaires were retained, corresponding to a valid response rate of 95.6%.

### 2.5. Statistical Analysis

All statistical analyses were performed using IBM SPSS Statistics for Windows, Version 23.0 (IBM Corp., Armonk, NY, USA). Continuous variables were expressed as mean ± standard deviation (M ± SD), and categorical variables were described as frequencies and percentages (%). Age was collected as a continuous variable and was treated as such in all regression analyses; the age categories presented in Table 1 were used solely for descriptive purposes. One-way analysis of variance (ANOVA) was used to compare differences in HPV vaccination willingness scores and Big Five personality traits across academic majors. For these between-group comparisons, the Bonferroni correction was applied to adjust for multiple post hoc tests.

Hierarchical multiple linear regression models were constructed to examine (a) the associations between Big Five personality traits and HPV vaccination willingness and (b) the moderating role of academic major in the extraversion–willingness relationship. Academic major was coded as a categorical variable with Preventive Medicine serving as the reference group, from which three dummy variables were generated: D1 (Nursing = 1, others = 0), D2 (Clinical Medicine = 1, others = 0), and D3 (Pharmacy = 1, others = 0). All continuous personality variables were mean-centered prior to analysis to reduce potential multicollinearity, and interaction terms were computed as the product of the centered extraversion score and each academic major dummy variable. The variance inflation factor (VIF) was examined to assess collinearity, with VIF values below 5 considered indicative of acceptable collinearity.

Four separate regression models were fitted, with consideration, determination, recommendation, and overall willingness serving as the dependent variables respectively. All models followed an identical three-step hierarchical procedure. In Step 1, demographic variables (age, academic year, residence, monthly living expenses, and sexual experience) and the three academic major dummy variables were entered to control for potential confounding. In Step 2, the centered scores of all five Big Five personality dimensions were added to examine the independent main effects of personality traits on vaccination willingness. In Step 3, three interaction terms (D1 × centered extraversion, D2 × centered extraversion, D3 × centered extraversion) were added to test whether academic major moderated the association between extraversion and vaccination willingness. Significant interactions were further probed using simple slope analyses to examine the effect of extraversion within each major group.

Given that the four dependent variables (consideration, determination, recommendation, and overall willingness) are conceptually related facets of a unified vaccination willingness construct and were analyzed within the same theoretical framework, no correction for multiple testing (e.g., Bonferroni) was applied across the four regression models. This decision was based on several considerations. First, the four outcome measures are not independent but represent strongly interrelated motivational dimensions, and applying a familywise error correction assumes independent tests, which is not the case here [35,36]. Second, as noted by Rothman (1990) and similarly by Goldberg and Silbergeld (2011), no adjustments are needed for multiple comparisons when the goal is to describe patterns of association rather than to test a universal null hypothesis [35,36]. Third, in exploratory analyses such as the present study, corrections for multiplicity can be overly conservative and substantially increase the risk of Type II error, potentially obscuring meaningful findings that warrant further investigation [37]. Fourth, the practice of using family-based error rates to make inferences about individual hypotheses has been criticized as logically inconsistent, because the evidentiary weight for a specific association should not depend on how many other associations were tested in the same study [38,39]. Consistent with these arguments, the interpretation of results emphasized the overall pattern and consistency of associations rather than isolated significant *p*-values [37]. All tests were two-sided, and a *p*-value of less than 0.05 was considered statistically significant.

## 3. Results

### 3.1. Participant Characteristics

The majority of participants were aged 20–21 years (54.2%). Of the 672 respondents, 193 (28.7%) majored in Nursing, 201 (29.9%) in Clinical Medicine, 147 (21.9%) in Pharmacy, and 131 (19.5%) in Preventive Medicine. In terms of academic year, 204 (30.4%) were first-year students, 281 (41.8%) were second-year students, and 187 (27.8%) were third-year students. A majority reported a rural residence (66.0%). A total of 414 (61.6%) participants reported monthly living expenses of 1001 to 1500 CNY (equivalent to 143–215 USD). Finally, 599 (89.1%) participants reported no sexual experience (Table 1).

### 3.2. Big Five Personality Traits of Participants

Participants reported moderately high levels of agreeableness (10.74 ± 1.79) and openness (7.05 ± 1.38) dimensions of the Big Five, with moderate scores observed for extraversion (6.22 ± 1.72), conscientiousness (6.20 ± 1.42), and neuroticism (6.15 ± 1.61). One-way ANOVA was conducted to compare the Big Five personality dimensions across majors. A statistically significant between-group difference was detected only for extraversion (*F* = 5.269, *p* = 0.001). Bonferroni-corrected post hoc tests further indicated that undergraduates majoring in Preventive Medicine reported significantly higher extraversion scores compared with peers in the Nursing, Clinical Medicine, and Pharmacy groups (Table 2).

### 3.3. HPV Vaccination Willingness of Participants

Participants reported high consideration scores (4.13 ± 0.78) and moderately high scores on determination (3.92 ± 0.87), recommendation (3.96 ± 0.80), and overall willingness (12.01 ± 2.17). One-way ANOVA was conducted to compare the three facets and overall willingness across majors. Statistically significant between-group differences were detected for the determination facet (*F* = 4.374, *p* = 0.005) and overall willingness (*F* = 3.851, *p* = 0.009). Bonferroni-corrected post hoc tests further indicated that undergraduates majoring in Preventive Medicine reported significantly higher scores for both determination and overall willingness compared with peers in the Nursing group (Table 3).

### 3.4. Correlation Between Big Five Personality Traits and HPV Vaccination Willingness

Pearson correlation analysis revealed that extraversion, agreeableness, conscientiousness, and openness were each significantly but modest positive correlated with all three facets of HPV vaccination willingness and the overall willingness score (*p* < 0.01). In contrast, neuroticism showed no significant correlation with any willingness facet or the overall score (all *p* > 0.05) (Table 4).

### 3.5. Hierarchical Linear Regression of Big Five Personality Traits on HPV Vaccination Willingness

Prior to the regression analyses, one-way ANOVA had revealed that Preventive Medicine students scored significantly higher on extraversion than those in Nursing, Clinical Medicine, and Pharmacy. To determine whether the association between extraversion and HPV vaccination willingness differed by academic major, interaction terms between extraversion and the major dummy variables were included in the regression models. No interaction terms were constructed for agreeableness, conscientiousness, neuroticism, or openness, as no significant between-major differences had been observed on these traits.

Four separate hierarchical multiple regression models were fitted, with consideration, determination, recommendation, and overall willingness serving as the dependent variables, respectively. The main effects of the Big Five personality traits were evaluated at Step 2, after controlling for demographic covariates and academic major.

Regression diagnostics indicated that the four regression models met all underlying assumptions. The maximum VIF across all predictor variables was 2.187, well below the commonly accepted threshold of 5, indicating that multicollinearity did not unduly influence the parameter estimates. The Durbin–Watson statistic ranged from 1.925 to 1.980, falling within the acceptable range of 1.5–2.5, confirming the independence of residuals. Visual inspection of normal probability (P-P) plots suggested that the residuals were approximately normally distributed, and scatterplots of standardized residuals versus predicted values showed no systematic patterns, supporting the assumptions of homoscedasticity and linearity.

#### 3.5.1. Model with Consideration as the Dependent Variable

At Step 2, agreeableness (*β* = 0.193, *p* < 0.001) and openness (*β* = 0.079, *p* = 0.046) were significantly but modest positive associated with consideration. The Step 2 model explained 6.1% of the variance (ΔR^2^ = 0.061, Δ*F* = 8.762, *p* < 0.001) (Table 5).

#### 3.5.2. Model with Determination as the Dependent Variable

At Step 2, agreeableness (*β* = 0.113, *p* = 0.004) and conscientiousness (*β* = 0.096, *p* = 0.015) were significant, albeit modest, correlates of determination. Extraversion was also significant but modestly positive at Step 2 (*β* = 0.103, *p* = 0.010). The model accounted for 4.9% of the variance (ΔR^2^ = 0.049, Δ*F* = 7.078, *p* < 0.001) (Table 6).

#### 3.5.3. Model with Recommendation as the Dependent Variable

At Step 2, extraversion (*β* = 0.125, *p* = 0.002), agreeableness (*β* = 0.129, *p* = 0.001), conscientiousness (*β* = 0.102, *p* = 0.009), and neuroticism (*β* = 0.081, *p* = 0.036) were each significantly but modestly positively associated with recommendation. Step 2 explained 6.1% of the variance (ΔR^2^ = 0.061, Δ*F* = 8.837, *p* < 0.001) (Table 7).

#### 3.5.4. Model with Overall Willingness as the Dependent Variable

At Step 2, extraversion (*β* = 0.103, *p* = 0.009), agreeableness (*β* = 0.162, *p* < 0.001), conscientiousness (*β* = 0.096, *p* = 0.013), and neuroticism (*β* = 0.076, *p* = 0.048) were significantly but modestly positively associated with the overall willingness score. Step 2 explained 6.8% of the variance (ΔR^2^ = 0.068, Δ*F* = 10.113, *p* < 0.001) (Table 8).

#### 3.5.5. Interaction Effects Across Models

In all four models, the academic major × centered extraversion interaction terms (D1 × centered extraversion, D2 × centered extraversion, D3 × centered extraversion) entered at Step 3 were non-significant (all *p* > 0.05) (Table 5, Table 6, Table 7 and Table 8), indicating that academic major did not moderate the association between extraversion and any HPV vaccination willingness indicator. Consequently, the main effects reported above are based on the more parsimonious Step 2 models.

In summary, agreeableness was the most consistent correlate of HPV vaccination willingness, showing significant but modestly positive associations across all four indicators. Conscientiousness was associated with determination, recommendation, and overall willingness. Extraversion and neuroticism were associated with recommendation and overall willingness. Openness was uniquely associated with consideration. These associations were not moderated by academic major.

## 4. Discussion

This study examined the associations between Big Five personality traits and HPV vaccination willingness among female healthcare undergraduates in China, and further tested whether academic major moderated the extraversion–willingness relationship. Rather than relying solely on the composite willingness score, we analyzed three distinct motivational facets—consideration, determination, and recommendation—to capture the nuances of how personality traits may relate to different stages of the vaccination decision-making process. The findings revealed that different personality traits were associated with different facets, with agreeableness emerging as the most consistent correlate across all three indicators. No moderating effect of academic major was found. It should be noted that the observed associations, while statistically significant, were modest in magnitude (r = 0.10–0.21). These effect sizes are consistent with the broader literature on personality and health behaviors, in which personality traits are viewed as distal predispositions whose influence is often mediated by more proximal cognitive and social factors [18,40]. Accordingly, the practical value of these findings lies in informing population-level vaccination communication strategies rather than in individual-level prediction.

### 4.1. Personality Traits and Consideration

The consideration facet captures the initial, tentative stage of the vaccination decision-making process (“I will consider receiving the HPV vaccine”). In the present study, agreeableness and openness were significantly and positively associated with consideration.

Agreeableness reflects an enduring disposition toward maintaining harmonious interpersonal relationships, adhering to social norms, and considering the welfare of others [41]. Individuals high in agreeableness are intrinsically motivated to comply with altruistic health norms and trustworthy public health recommendations, which drives a positive cognitive appraisal of HPV vaccination. This interpretation is consistent with meta-analytic evidence identifying agreeableness as one of the most robust personality correlates of favorable vaccination attitudes across diverse samples and vaccine types [18]. For healthcare undergraduates, whose professional training frames vaccination as a responsible behavior aligned with both medical standards and societal expectations, this predisposition is likely reinforced, translating into more active consideration of the HPV vaccine.

Openness reflects the openness, wisdom, and creativity of individuals to experience (i.e., creativity, curiosity, and willingness to accept new ideas) [42]. Individuals high in openness are more receptive to new ideas and experiences [43]. Extensive literature indicates a positive correlation between openness and willingness to receive the COVID-19 vaccine [44,45,46]; this is likely because open individuals tend to perceive vaccination as a new experience that they are more willing to accept [44,46]. Given that the introduction of the HPV vaccine in China lagged behind many Western countries, HPV vaccination may still be perceived as relatively new among young Chinese women. Indeed, Zhang et al. (2025) also demonstrated that Chinese residents with higher openness exhibited greater acceptance of the HPV vaccine [26].

However, the participants in the present study were female healthcare undergraduates; within their professional context, the HPV vaccine is not considered a novelty. Therefore, the observed positive association between openness and vaccination consideration should instead be interpreted through the lens of receptiveness to new ideas. In China and broader Asian contexts, conservative sexual morals and the stigmatization of HPV as a sexually transmitted infection pose distinct barriers to HPV vaccination [5,47,48]. Against this backdrop, a positive consideration of the HPV vaccine can be viewed as a progressive idea that challenges tradition. In this sense, the willingness to consider vaccination represents not merely a health decision but also a cognitive departure from conservative norms that stigmatize the vaccine. Highly open individuals are more capable of embracing such ideas, resisting the restrictive influences of conservative sexual morals and stigma, and instead relying on scientific evidence to make independent health decisions. Notably, as neither sexual norms nor stigmatization were directly measured in this study, the proposed interpretation regarding openness as a buffer against socio-cultural barriers remains hypothetical and warrants direct empirical testing.

### 4.2. Personality Traits and Determination

The determination facet captures a firm commitment to receiving the HPV vaccine (“I will definitely receive the HPV vaccine”), representing a more advanced stage of the motivational process in which an individual has moved beyond initial consideration to a concrete behavioral intention. In the present study, extraversion, agreeableness, and conscientiousness were significantly and positively associated with determination at Step 2 of the regression model.

The pattern of significant correlates can be understood through the lens of decision-making styles. Scott and Bruce (1995) proposed five distinct decision-making styles: rational, intuitive, dependent, avoidant, and spontaneous [49]. Some research has found an association between personality traits and decision-making styles [50,51]. In a study of medical students specifically, El Othman et al. (2020) found that extraversion was positively associated with intuitive decision-making, agreeableness with dependent decision-making, and conscientiousness with rational decision-making [52]. These associations provide a framework for interpreting the present findings. However, as decision-making styles were not directly assessed in this study, the following interpretations remain theoretical and should be tested in future research incorporating validated measures of decision-making styles.

Regarding extraversion, individuals high in this trait tend to adopt an intuitive decision-making style, characterized by a reliance on feelings, hunches, and rapid pattern recognition rather than exhaustive deliberation [49,52]. In the context of HPV vaccination decisions, such an intuitive—and seemingly irrational—approach might appear incongruent with the rigorous scientific training received by healthcare students. However, intuition and rationality are not necessarily opposing or mutually exclusive. Cert and Wilcockson (1996) argued that the dichotomy between intuition and rationality in professional thinking is a false one, contending that intuition in professional contexts is “a non-rational process which has a rational basis,” grounded in a solid foundation of relevant knowledge rather than operating independently of it [53]. In the medical context, Jacobs (2025) [54] argued that intuition is a product of pre-conscious predictive processing based on implicit professional knowledge. It has an indicative function and motivational properties, and serves as a reliable resource for clinical reasoning rather than mere guesswork [54]. For the healthcare students in the present sample, knowledge regarding the safety, efficacy, and public health importance of the HPV vaccine is systematically reinforced throughout their professional curricula. Under these conditions, the intuitive decision-making style associated with extraversion may operate as a cognitively efficient pathway: extraverted individuals may draw upon internalized professional knowledge to make decisive vaccination decisions through intuition. It should be noted, however, that professional intuition was not directly measured in this study; this interpretation is therefore a theoretical account that requires empirical verification in studies specifically designed to assess intuitive decision-making in medical contexts.

Regarding agreeableness, individuals high in this trait tend to adopt a dependent decision-making style, characterized by a willingness to seek advice, trust external guidance, and rely on the recommendations of others when making important decisions [49,52]. In the context of HPV vaccination, this dependent style is likely to manifest as a trust-based acceptance of professional medical and public health recommendations. For healthcare students specifically, this pathway is reinforced by their professional education, which not only provides scientific knowledge about vaccines but also cultivates an understanding of the institutional authority and evidentiary standards underpinning public health guidelines. Agreeable individuals’ innate tendency to trust [43], combined with their professional appreciation of the validity of those recommendations, may thus help translate positive attitudes into resolute decisions.

Regarding conscientiousness, individuals high in this trait tend to favor a rational decision-making style, approaching important choices through systematic information gathering, careful evaluation of alternatives, and logical deliberation [49,52]. In the context of HPV vaccination, this rational approach is likely to involve a thorough assessment of the vaccine’s safety, efficacy, and personal and public health benefits. For healthcare students, their professional training equips them with the knowledge base and analytical framework to evaluate medical evidence. Consequently, the rational decision-making pathway is particularly accessible to them in forming a resolute vaccination decision.

### 4.3. Personality Traits and Recommendation

The recommendation facet is distinctive in that it extends beyond personal health decisions to involve active peer advocacy (“I will recommend the HPV vaccine to my friends or classmates”). At Step 2, extraversion, agreeableness, conscientiousness, and neuroticism were each significantly and positively associated with recommendation.

Extraversion is characterized by sociability [43]. Sociability predisposes extraverted individuals to seek out and enjoy interpersonal interactions, making them more likely to initiate conversations about health topics and offer advice to peers. For healthcare students specifically, this social orientation is complemented by their professional knowledge: their medical training provides the substantive basis for an informed recommendation, while extraversion supplies the interpersonal impetus to actively deliver it.

Agreeableness is characterized by altruism [43]. Altruism reflects a genuine concern for the welfare of others, providing a direct motivational basis for recommendation behavior. For healthcare students specifically, this altruistic motivation is reinforced by their professional education. Agreeable healthcare students may therefore be particularly active in peer advocacy.

Conscientiousness is characterized by a tendency toward responsibility and adherence to rules and norms [43]. A strong sense of responsibility and compliance with rules lead conscientious individuals to consistently maintain healthy lifestyles [55]. They may also rationally offer health advice to others based on rigorous scientific evidence or clear guidelines, motivated by their sense of social responsibility. Given that public health authorities and medical organizations explicitly recommend HPV vaccination for young women, conscientious healthcare students not only comply with these guidelines themselves but also encourage others to do so. Their recommendation behavior can therefore be understood as an expression of norm adherence in the health domain.

Furthermore, existing research indicates that these three personality traits are positively associated with knowledge sharing [56,57,58]. This provides further empirical support for the significant positive correlation observed between these traits and recommendation.

Notably, the significant positive association between neuroticism and recommendation stands in contrast to the wider vaccination literature, in which neuroticism is generally linked to either negative or null associations with vaccination attitudes and behaviors [18]. However, the research of Weiss and Deary (2020) offers a sound explanatory framework, which distinguishes between “worried–vulnerable” neuroticism and “anxious–tense” neuroticism [59]. Worried–vulnerable neuroticism, characterized by health-related vigilance and concern about physical vulnerability, has been linked to greater engagement in health-promoting behaviors [59]. In the present sample of female healthcare students, who possess professional medical knowledge that allows for a more scientific consideration of health issues, neuroticism might be expressed as heightened vigilance regarding health risks instead of mere anxiety or tension. This vigilance may extend beyond concerns for their own health to encompass the well-being of others within their social networks. In the context of HPV vaccination, they are highly vigilant about the risk of others contracting HPV. Driven by medical professional ethics, they may actively offer recommendations to others based on their expertise.

It is worth noting that neuroticism was not significantly correlated with vaccination willingness in bivariate analyses, yet emerged as a significant predictor in the regression model for recommendation. This pattern suggests a possible statistical suppression effect. Specifically, the inclusion of other personality traits, particularly conscientiousness and agreeableness which tend to correlate negatively with neuroticism, may have controlled for variance in neuroticism that is unrelated or negatively related to vaccination willingness, thereby revealing its unique positive association with peer recommendation. However, as neuroticism and its facets were not directly assessed with specific measures, this interpretation requires future replication and validation. Furthermore, the mechanisms proposed here, including health-related vigilance and the worried–vulnerable subtype, are theoretical interpretations that should be tested in future research using dedicated measures of health anxiety and neuroticism facets.

### 4.4. Personality Traits and Overall Willingness

Although the primary focus of this study was on the three motivational facets, the results for the overall HPV vaccination willingness score were largely consistent with the facet-level findings. Across all four indicators, agreeableness emerged as the most consistent and robust correlate, showing significant positive associations with consideration, determination, recommendation, and overall willingness. This pattern is consistent with the meta-analytic finding that agreeableness is one of the most robust personality predictors of positive vaccination attitudes across diverse samples and vaccine types [18].

However, the specific pattern of associations observed in this study differs from findings reported in some other studies of HPV vaccination. For instance, in a nationwide survey in China, Zhang et al. (2025) found that extraversion, openness, and conscientiousness—but not agreeableness—were significantly associated with HPV vaccination behavior [26]. Several factors may account for this discrepancy. First, the present sample comprised female healthcare undergraduates, whereas Zhang et al. (2025) [26] examined a general population with diverse educational and occupational backgrounds. Second, the present study assessed vaccination willingness, whereas Zhang et al. (2025) [26] measured actual vaccination behavior. Third, the two studies employed different statistical approaches. Given these differences in population, outcome measurement, and methodology, the divergent findings are not necessarily contradictory but rather reflect the context-dependent nature of personality–vaccination associations.

More broadly, the findings of this study both align with and diverge from the broader vaccination literature in ways that illustrate the complexity of personality–vaccination relationships. Bleidorn et al. (2025) noted that different personality traits may be linked to different vaccination outcomes, and sociodemographic and cultural differences across studies might also have contributed to the mixed state of evidence [18]. The present findings lend empirical weight to this observation and underscore the importance of contextualizing personality–vaccination associations within specific population characteristics.

### 4.5. The Non-Significant Moderating Effect of Academic Major

One novel aspect of this study was the examination of whether academic major moderated the association between extraversion and HPV vaccination willingness. This analysis was prompted by the finding from the one-way ANOVA that Preventive Medicine students scored significantly higher on extraversion than students in Nursing, Clinical Medicine, and Pharmacy. To test this possibility, interaction terms between extraversion and the three academic major dummy variables were included in Step 3 of the hierarchical regression models.

Across all four willingness indicators, no interaction between extraversion and academic major attained statistical significance. In models predicting determination, recommendation and overall willingness, extraversion showed significant but modest positive associations in Step 2 but turned non-significant after interaction terms were included in Step 3. This shift was likely attributable to greater model complexity, reduced degrees of freedom and lowered statistical power brought by non-significant interaction terms, rather than unstable main effects. Thus, Step 2 outcomes represent more parsimonious estimates of the association between extraversion and vaccination willingness.

The absence of significant moderation suggests that the positive association between extraversion and vaccination willingness was stable across academic majors. Whether a student was enrolled in Preventive Medicine, Nursing, Clinical Medicine, or Pharmacy did not alter the degree to which extraversion was associated with greater willingness to consider, commit to, or recommend HPV vaccination. This null finding should be interpreted within the context of the sample characteristics. All participants were students at the same medical college, shared a common core curriculum in medicine and public health, and were socialized within a similar professional environment. This shared professional knowledge base and similar exposure to health information may have attenuated any potential differences in how personality traits are associated with vaccination decision-making across different majors. Future research with larger and more diverse samples across multiple institutions and healthcare disciplines would be valuable to further examine whether and how professional training contexts moderate the links between personality traits and health-related decision-making.

### 4.6. Limitations

Several limitations of the present study should be noted. First, the cross-sectional design cannot determine causal relationships between Big Five personality traits and HPV vaccination willingness.

Second, the study was conducted at a single public independent medical college in Chengdu, China, and the sample comprised exclusively female healthcare undergraduates who had previously heard of HPV and the HPV vaccine. This limits the generalizability of the findings in several respects. Specifically, students at a single institution may share similar educational experiences, regional cultural norms, and socioeconomic backgrounds, which may not be representative of medical students in other parts of China or in non-medical institutions. Moreover, as healthcare students, participants possess above-average health literacy compared to the general population, which may further limit the applicability of the findings to women with lower health literacy or different educational backgrounds. Furthermore, the inclusion of only female students means the findings cannot be generalized to male students or to young men who are also eligible for HPV vaccination. Finally, all participants reported having heard of both HPV and the HPV vaccine, indicating a baseline level of awareness that may not be present in the broader population of young women. As personality traits may interact differently with vaccination decision-making among individuals with limited HPV-related knowledge, the results should not be extended to populations unfamiliar with the vaccine. Future multi-center, cross-regional studies with more diverse samples (e.g., including male students, non-medical students, and individuals with varying levels of HPV awareness) are needed to assess the broader applicability of these findings.

Third, this study only examined academic major as a moderating factor and did not consider other situational variables that may influence the association between personality traits and vaccination willingness.

Fourth, the internal consistency of the BFI-10 subscales was relatively low in this sample. While supplementary reliability indicators (inter-item correlations and Spearman–Brown coefficients) were within ranges expected for ultra-brief measures, measurement error may have attenuated the observed effect sizes and increased the risk of Type II error. Furthermore, the 11-item version of the BFI-10 used in this study, which included a supplementary agreeableness item as recommended by the original developers [21], has not been formally validated in Chinese healthcare student populations. Recent cross-national studies have raised concerns about the BFI-10’s structural properties [60,61]. However, emerging evidence suggests that these psychometric challenges primarily stem from translation errors and cross-cultural sampling variability rather than from a fundamental flaw in the instrument itself [62,63]. Although the BFI-10 offers advantages in time-constrained survey settings and its reliability has been supported to some degree [63,64,65], current recommendations for personality assessment in health research advocate for the use of longer, facet-level instruments when feasible, as these enable more nuanced investigation of personality–health associations [40,65]. Methodologists have also called for incorporating multi-source data beyond single-method self-reports to enhance the robustness of findings [66]. Future research should prioritize comprehensive personality measures when feasible, ensure rigorous translation and measurement equivalence testing in cross-national studies, and incorporate multi-source data to strengthen validity. In addition, future studies should formally validate the 11-item BFI-10 in Chinese healthcare student populations.

Fifth, each facet of HPV vaccination willingness was measured using a single item, which inevitably introduces greater measurement error compared to multi-item scales. In the regression analyses, this measurement error may have attenuated the observed associations between personality traits and specific willingness facets, reducing statistical power and increasing the risk of Type II error. Consequently, some personality–willingness associations may have gone undetected at the facet level. Future research should employ multi-item scales for each facet to replicate and extend these findings with higher psychometric precision.

Sixth, the regression models explained a modest proportion of the variance in HPV vaccination willingness. While these effect sizes are consistent with the small-to-medium effects typically observed in research on personality and vaccine attitudes and behaviors [18], they indicate that a substantial portion of variance in vaccination willingness is attributable to factors not measured in the present study. Personality traits are distal determinants of health behavior that are theorized to exert their influence through more proximal psychological mechanisms, including vaccine-specific knowledge, risk perceptions, perceived susceptibility and severity, and social norms. Moreover, practical and structural factors—such as vaccine cost, accessibility, and family support—may play equally or more important roles in vaccination decisions. Future research should integrate personality traits with established health behavior frameworks to examine how distal personality dispositions interact with proximal cognitive and social determinants of vaccination decisions.

Seventh, all data were collected via self-report questionnaires, which may introduce subjective reporting bias.

Finally, the three facet scores of HPV vaccination willingness were derived from single Likert-type items and treated as continuous variables in the regression analyses, in line with common practice in health behavior research [67]. Nonetheless, future research could employ ordinal regression models as a sensitivity analysis to further confirm the robustness of these findings.

## 5. Conclusions

This study examined the relationships between Big Five personality traits and HPV vaccination willingness among female healthcare undergraduates. The analyses identified unique trait correlates for each facet of vaccination intention. Specifically, agreeableness and openness were positively correlated with vaccination consideration; agreeableness and conscientiousness were positively correlated with vaccination determination; and extraversion, agreeableness, conscientiousness, and neuroticism were positively correlated with peer vaccination recommendation. No significant moderating effect of academic major was observed on the association between extraversion and the various facets of vaccination intention. The observed associations, while statistically significant, were modest in magnitude.

These findings offer several practical implications for vaccination promotion. Health communication strategies emphasizing the altruistic and communal benefits of HPV vaccination may resonate broadly, particularly given the consistent association between agreeableness and willingness across all facets. Information-based interventions designed to dispel culturally embedded misconceptions and stigma may facilitate initial consideration, especially among individuals lower in openness. Furthermore, framing vaccination as a responsible, evidence-based health behavior supported by clear professional guidelines may strengthen commitment among conscientious individuals. For peer recommendation, empowering healthcare students with accurate knowledge and communication skills may enhance their capacity to serve as informal vaccination advocates within their social networks.

Several avenues for future research warrant exploration. Longitudinal designs are required to clarify whether the identified personality correlates translate into actual vaccination uptake. Furthermore, multi-center studies involving diverse cohorts, including male and non-medical students as well as individuals with varying HPV awareness, are necessary to assess the broader generalizability of these findings.

In conclusion, this study demonstrates that stable personality dispositions are differentially associated with distinct stages of HPV vaccination decision-making among future health professionals. While the observed effect sizes were modest, these findings may inform the development of personality-sensitive vaccination communication strategies and highlight avenues for further investigation into the psychological determinants of vaccine acceptance.

## Figures and Tables

**Table 1:** Demographic characteristics of participants (*n* = 672).

Variable	Group	Frequency (*n*)	Percent (%)
Age	① ≤19	244	36.3
	② 20~21	364	54.2
	③ ≥22	64	9.5
Academic Major	① Nursing	193	28.7
	② Clinical Medicine	201	29.9
	③ Pharmacy	147	21.9
	④ Preventive Medicine	131	19.5
Academic year	① 1st	204	30.4
	② 2nd	281	41.8
	③ 3rd	187	27.8
Residence	Urban	235	35.0
	Rural	437	65.0
Monthly Living Expenses (CNY)	① ≤1000	58	8.6
	② 1001~1500	414	61.6
	③ 1501~2000	177	26.3
	④ ≥2001	23	3.4
Sexual experience	Yes	73	10.9
	No	599	89.1

**Table 2:** Descriptive statistics and one-way ANOVA results for Big Five personality traits by major (M ± SD).

Variable	Extraversion	Agreeableness	Conscientiousness	Neuroticism	Openness
1. Nursing	6.10 ± 1.70	10.58 ± 1.87	6.16 ± 1.28	6.23 ± 1.58	6.92 ± 1.46
2. Clinical Medicine	6.14 ± 1.80	10.77 ± 1.85	6.16 ± 1.33	6.15 ± 1.65	7.11 ± 1.36
3. Pharmacy	6.01 ± 1.66	10.82 ± 1.68	6.05 ± 1.50	6.16 ± 1.53	7.05 ± 1.27
4. Preventive Medicine	6.74 ± 1.60	10.86 ± 1.68	6.46 ± 1.60	6.05 ± 1.70	7.15 ± 1.41
Total	6.22 ± 1.72	10.74 ± 1.79	6.20 ± 1.42	6.15 ± 1.61	7.05 ± 1.38
*F*	5.269	0.860	2.071	0.334	0.940
*p*	0.001	0.461	0.103	0.800	0.421
Bonferroni post hoc test	4 > 1,2,3	-	-	-	-

**Table 3:** Descriptive statistics and one-way ANOVA results for HPV vaccination willingness by major.

Variable	Consideration	Determination	Recommendation	WILL_O_
1. Nursing	4.03 ± 0.81	3.76 ± 0.88	3.83 ± 0.84	11.62 ± 2.28
2. Clinical Medicine	4.14 ± 0.82	3.93 ± 0.89	4.01 ± 0.77	12.08 ± 2.14
3. Pharmacy	4.14 ± 0.71	3.93 ± 0.84	3.96 ± 0.78	12.02 ± 2.10
4. Preventive Medicine	4.27 ± 0.73	4.11 ± 0.84	4.05 ± 0.79	12.44 ± 2.08
Total	4.13 ± 0.78	3.92 ± 0.87	3.96 ± 0.80	12.01 ± 2.17
*F*	2.434	4.374	2.609	3.851
*p*	0.064	0.005	0.051	0.009
Bonferroni post hoc test	-	4 > 1	-	4 > 1

Note: WILL_O_ = Overall Willingness.

**Table 4:** Correlations of Big Five personality traits with HPV vaccination willingness and its facets (R-value).

Variable	Consideration	Determination	Recommendation	WILL_O_
Extraversion	0.109 **	0.168 **	0.178 **	0.172 **
Agreeableness	0.214 **	0.146 **	0.162 **	0.195 **
Conscientiousness	0.110 **	0.146 **	0.149 **	0.153 **
Neuroticism	0.002	−0.001	0.012	0.005
Openness	0.155 **	0.132 **	0.126 **	0.154 **

Note: WILL_O_ = Overall Willingness. ** *p* < 0.01.

**Table 5:** Hierarchical regression: Big Five personality traits on consideration (Moderated by Major) (*β*-value).

Variables	Consideration			
	Layer 1	Layer 2	Layer 3	95% CI
Age	0.040	0.036	0.033	[−0.038, 0.104]
Academic year	0.012	0.003	−0.003	[−0.113, 0.107]
Residence	−0.023	−0.017	−0.014	[−0.141, 0.113]
Monthly Living Expenses	0.126 **	0.115 **	0.121 **	[0.025, 0.217]
Sexual experience	0.046	0.041	0.041	[−0.147, 0.229]
D1	−0.113 *	−0.087	−0.080	[−0.256, 0.096]
D2	−0.080	−0.062	−0.051	[−0.223, 0.121]
D3	−0.059	−0.043	−0.037	[−0.221, 0.147]
Centered Extraversion		0.045	0.108	[0.026, 0.190]
Centered Agreeableness		0.193 **	0.191 **	[0.158, 0.224]
Centered Conscientiousness		0.056	0.058	[0.015, 0.101]
Centered Neuroticism		0.060	0.058	[0.021, 0.095]
Centered Openness		0.079 *	0.085 *	[0.042, 0.128]
D1 × Centered Extraversion			−0.084	[−0.186, 0.018]
D2 × Centered Extraversion			0.007	[−0.093, 0.107]
D3 × Centered Extraversion			−0.049	[−0.159, 0.061]
*F*	2.469 *	4.979 **	4.293 **	-
△*F*	-	8.762 **	1.294	-
R^2^	0.029	0.090	0.095	-
△R^2^	-	0.061	0.005	-

* *p* < 0.05, ** *p* < 0.01.

**Table 6:** Hierarchical regression: Big Five personality traits on determination (Moderated by Major) (β-value).

Variables	Determination			
	Layer 1	Layer 2	Layer 3	95% CI
Age	−0.061	−0.067	−0.069	[−0.147, 0.009]
Academic year	−0.103	−0.116	−0.121	[−0.243, 0.001]
Residence	−0.047	−0.040	−0.039	[−0.182, 0.104]
Monthly Living Expenses	0.130 **	0.111 **	0.115	[0.007, 0.223]
Sexual experience	0.008	0.014	0.017	[−0.195, 0.229]
D1	−0.150 **	−0.117 *	−0.116	[−0.312, 0.080]
D2	−0.092	−0.063	−0.061	[−0.253, 0.131]
D3	−0.068	−0.039	−0.043	[−0.249, 0.163]
Centered Extraversion		0.103 *	0.103	[0.011, 0.195]
Centered Agreeableness		0.113 **	0.114 **	[0.077, 0.151]
Centered Conscientiousness		0.096 *	0.098 *	[0.051, 0.145]
Centered Neuroticism		0.061	0.057	[0.016, 0.098]
Centered Openness		0.055	0.060	[0.011, 0.109]
D1 × Centered Extraversion			−0.010	[−0.124, 0.104]
D2 × Centered Extraversion			0.040	[−0.072, 0.152]
D3 × Centered Extraversion			−0.044	[−0.166, 0.078]
*F*	4.189 **	5.418 **	4.563 **	-
△*F*	-	7.078 **	0.874	-
R^2^	0.048	0.097	0.100	-
△R^2^	-	0.049	0.004	-

* *p* < 0.05, ** *p* < 0.01.

**Table 7:** Hierarchical regression: Big Five personality traits on recommendation (Moderated by Major) (β-value).

Variables	Recommendation			
	Layer 1	Layer 2	Layer 3	95% CI
Age	−0.097	−0.103	−0.106	[−0.177, −0.035]
Academic year	−0.113	−0.125	−0.131	[−0.243, −0.019]
Residence	−0.006	0.000	0.002	[−0.129, 0.133]
Monthly Living Expenses	0.135 **	0.115 **	0.121 **	[0.023, 0.219]
Sexual experience	0.043	0.051	0.053	[−0.139, 0.245]
D1	−0.086	−0.049	−0.048	[−0.226, 0.130]
D2	−0.016	0.017	0.021	[−0.155, 0.197]
D3	−0.027	0.006	0.004	[−0.184, 0.192]
Centered Extraversion		0.125 **	0.134	[0.050, 0.218]
Centered Agreeableness		0.129 **	0.129 **	[0.096, 0.162]
Centered Conscientiousness		0.102 **	0.105 **	[0.062, 0.148]
Centered Neuroticism		0.081 *	0.077 *	[0.040, 0.114]
Centered Openness		0.047	0.053	[0.008, 0.098]
D1 × Centered Extraversion			−0.037	[−0.141, 0.067]
D2 × Centered Extraversion			0.044	[−0.058, 0.146]
D3 × Centered Extraversion			−0.039	[−0.151, 0.073]
*F*	3.317 **	5.561 **	4.727 **	-
△*F*	-	8.837 **	1.105 **	-
R^2^	0.038	0.099	0.104	-
△R^2^	-	0.061	0.005	-

* *p* < 0.05, ** *p* < 0.01.

**Table 8:** Hierarchical regression: Big Five personality traits on overall willingness (Moderated by Major) (β-value).

Variables	Overall Willingness			
	Layer 1	Layer 2	Layer 3	95% CI
Age	−0.045	−0.052	−0.055	[−0.249, 0.139]
Academic year	−0.079	−0.091	−0.097	[−0.399, 0.205]
Residence	−0.030	−0.022	−0.020	[−0.375, 0.335]
Monthly Living Expenses	0.147 **	0.128 **	0.134 **	[−0.131, 0.399]
Sexual experience	0.035	0.039	0.041	[−0.480, 0.562]
D1	−0.133 *	−0.096	−0.092	[−0.576, 0.392]
D2	−0.071	−0.041	−0.035	[−0.509, 0.439]
D3	−0.058	−0.029	−0.029	[−0.537, 0.479]
Centered Extraversion		0.103 **	0.129	[−0.098, 0.356]
Centered Agreeableness		0.162 **	0.161 **	[0.069, 0.253]
Centered Conscientiousness		0.096 *	0.098 *	[−0.020, 0.216]
Centered Neuroticism		0.076 *	0.072	[−0.030, 0.174]
Centered Openness		0.067	0.074	[−0.048, 0.196]
D1 × Centered Extraversion			−0.048	[−0.330, 0.234]
D2 × Centered Extraversion			0.035	[−0.239, 0.309]
D3 × Centered Extraversion			−0.050	[−0.350, 0.250]
*F*	3.926 **	6.471 **	5.496 **	-
△*F*	-	10.113 **	1.239 *	-
R^2^	0.045	0.113	0.118	-
△R^2^	-	0.068	0.005	-

* *p* < 0.05, ** *p* < 0.01.

## Data Availability

The original data can be obtained by contacting the corresponding author.

## Supplementary Materials

The following supporting information can be downloaded at: https://www.mdpi.com/article/10.3390/vaccines14070610/s1, Example of the questionnaire.

## Abbreviations

HPV	Human Papillomavirus
BFI-10	10-item Big Five Inventory
QR	Quick Response
CNY	China Yuan
ANOVA	Analysis of Variance
VIF	Variance Inflation Factor
