# Supplementary material for: The Impact of Big Five Personality Traits on HPV Vaccination Willingness Among Female Healthcare Undergraduates: A Cross-Sectional Study in Chengdu, China
Source: Vaccines (Basel). 2026 Jul 11;14(7):610. doi: 10.3390/vaccines14070610 (PMC13417279; doi:10.3390/vaccines14070610)
Supplement: Supplementary file 1 [file vaccines-14-00610-s001.zip › vaccines-4389398-supplementary.pdf]

Example of the questionnaire

Title: Personality traits and HPV vaccination willingness among female healthcare undergraduates

Part 1: Demographic Characteristics Questionnaire

1. Age: ______ years old
2. Academic major:
○ Nursing
○ Clinical Medicine
○ Pharmacy
○ Preventive Medicine
3. Academic year:
○ 1st year
○ 2nd year
○ 3rd year
4.Residence:
○ Urban
○ Rural
5. What is your average monthly living expense? (CNY):
○ ≤ 1,000
○ 1,001 – 1,500
○ 1,501 – 2,000
○ > 2,000
6. Have you had sexual experience?
○ Yes
○ No

Part 2: Personality Questionnaire (BFI-10 with an additional agreeableness item)

How well do the following statements describe your personality?

**Instructions**: How well do the following statements describe your personality? I see myself as someone who …	Disagree strongly	Disagree a little	Neither agree nor disagree	Agree a little	Agree strongly
1. … is reserved	(1)	(2)	(3)	(4)	(5)
2. … is generally trusting	(1)	(2)	(3)	(4)	(5)
3. … tends to be lazy	(1)	(2)	(3)	(4)	(5)
4. … is relaxed, handles stress well	(1)	(2)	(3)	(4)	(5)
5. … has few artistic interests	(1)	(2)	(3)	(4)	(5)
6. … is outgoing, sociable	(1)	(2)	(3)	(4)	(5)
7. … tends to find fault with others	(1)	(2)	(3)	(4)	(5)
8. … does a thorough job	(1)	(2)	(3)	(4)	(5)

9. … gets nervous easily	(1)	(2)	(3)	(4)	(5)
10. … has an active imagination	(1)	(2)	(3)	(4)	(5)
11. …is considerate and kind to almost everyone.	(1)	(2)	(3)	(4)	(5)

Part 3: HPV Vaccination Willingness Scale

**Statement**	Disagree strongly	Disagree	Neutral	Agree	Agree strongly
1. I will consider receiving the HPV vaccine	(1)	(2)	(3)	(4)	(5)
2. I will definitely receive the HPV vaccine.	(1)	(2)	(3)	(4)	(5)
3. I will recommend the HPV vaccine to my friends or classmates.	(1)	(2)	(3)	(4)	(5)
